# Physical Performance Differences Across Competitive Stages in Brazilian Women’s Football: A Capacity-Specific Analysis from Under-14 to Professional Players

**DOI:** 10.3390/sports14090400

**Published:** 2026-09-11

**Authors:** Artur Avelino Birk Preissler, Pedro Schons, Filipe Manuel Clemente, Ana Filipa Silva, Carla Gonçalves, Luiz Fernando Martins Kruel

**Affiliations:** 1 Faculdade SOGIPA, Porto Alegre 90240-485, RS, Brazil; pedroschons@hotmail.com; 2 School of Physical Education, Physiotherapy and Dance, Federal University of Rio Grande do Sul, Porto Alegre 90690-200, RS, Brazil; kruel@esef.ufrgs.br; 3 Polytechnic University of Coimbra, 3045-093 Coimbra, Portugal; filipe.clemente5@gmail.com; 4 Sport Physical Activity and Health Research & Innovation Center, Applied Research Institute, Polytechnic University of Coimbra, 3045-093 Coimbra, Portugal; 5 Gdansk University of Physical Education and Sport, 80-336 Gdańsk, Poland; 6 Faculty of Sport Sciences and Physical Education, University of Coimbra, 3040-256 Coimbra, Portugal; anafilsilva@gmail.com; 7 Escola Superior de Desporto e Lazer, Universidade Politécnica de Viana do Castelo, 4900-347 Viana do Castelo, Portugal; carlagoncalves@esdl.ipvc.pt; 8 The Sport Physical Activity and Health Research & Innovation Center, Universidade Politécnica de Viana do Castelo, 4900-347 Viana do Castelo, Portugal

**Keywords:** soccer, physical fitness, adolescent, adult, exercise test, running

## Abstract

Physical performance may vary across competitive categories in women’s football, but the capacities distinguishing consecutive stages remain unclear. This cross-sectional study compared under-14 (U14), under-16 (U16), under-18 (U18), and professional Brazilian female outfield players and identified adjacent-stage differences. Overall, 119 female outfield players from two Brazilian football clubs, with U14 and U16 players recruited from one club and U18 and professional players from both clubs, completed the squat jump, countermovement jump, 20 m sprint, 20 m change-of-direction test (COD-20m), and Yo-Yo Intermittent Recovery Test Level 1. Analyses included Welch’s ANOVA, multivariate analysis, Games–Howell tests, and adjacent-stage comparisons with Holm adjustment. The multivariate physical-performance profile differed in all three adjacent-stage comparisons (Holm-adjusted *p* ≤ 0.043). U16 covered 491.82 m more than U14 in the Yo-Yo test (adjusted *p* = 0.012), although this comparison was limited to players from one club. U18 exceeded U16 in squat jump (3.10 cm; adjusted *p* = 0.004) and countermovement jump (3.87 cm; adjusted *p* = 0.002). Professionals exceeded U18 in squat jump (2.89 cm; adjusted *p* = 0.008), countermovement jump (2.93 cm; adjusted *p* = 0.011), and COD-20m (−0.24 s; adjusted *p* < 0.001). These category- and capacity-specific differences support category-specific interpretation and may help practitioners prioritize relevant capacities in monitoring and physical preparation rather than applying uniform benchmarks.

## 1. Introduction

Women’s football is an intermittent, multidirectional sport in which prolonged low- and moderate-intensity activity is repeatedly interrupted by high-speed running, sprinting, accelerations, decelerations, and other explosive actions [1,2,3,4]. These demands are position specific and may vary according to the tactical and locomotor requirements of playing roles, and can decline toward the end of the match, particularly for high-speed running and intense acceleration–deceleration activity [2,5]. Across the developmental spectrum, match-running performance generally increases with playing standard, and research involving Brazilian national teams indicates greater high-intensity and sprint activity in senior than in U17 and U20 players [6,7]. These observations make the development and monitoring of intermittent endurance and neuromuscular qualities relevant throughout the pathway to senior football [8].

In practical contexts, lower-limb power, acceleration and sprint speed, change-of-direction performance, and intermittent running capacity are commonly assessed using field-based tests that can be integrated within routine monitoring [8,9]. The squat jump (SJ) and countermovement jump (CMJ) are some of the popular neuromuscular tests, expressing vertical-jump performance and being used for different interpretations [10,11]. Short linear sprints primarily assess acceleration over shorter distances, whereas longer sprint distances are required to better characterize maximal sprint speed [8]. The Yo-Yo Intermittent Recovery Test Level 1 (Yo-Yo IRL1) assesses the ability to repeatedly perform intense intermittent exercise until exhaustion [12]. Interpretation is more complex in adolescent players because chronological age, biological maturation, body size, and accumulated training can influence these qualities to different degrees [13,14,15]. This evidence suggests that the same physical measures may not be equally informative at early-adolescent, late-adolescent, and senior stages [9,16].

The most recent meta-analysis in women’s football found progressively better intermittent aerobic capacity, sprint performance, and lower-limb power across higher performance tiers, while also showing that the magnitude of between-tier differences depended on the test and protocol used [9]. Some studies comparing age categories likewise show that older or senior female players are generally better than younger players in sprinting, jumping, change of direction, or intermittent fitness, although the tendency is neither uniform nor strictly linear across capacities [16,17,18]. For example, U16 and U18 players have shown faster sprint performance than U14 players without meaningful differences between U16 and U18, and maturity status has been associated with speed, change of direction, jump, and aerobic outcomes in elite youth players [13,14]. A longitudinal national team program further showed that developmental trajectories differed for sprinting, SJ, CMJ, and intermittent fitness, reinforcing the view that physical development is multidimensional [16]. In Brazilian national teams, U20 and senior players have shown advantages in lower-limb power and other fitness characteristics over younger categories, while match-running activity has generally increased from U17 to U20 and senior competition [7,19].

Despite this direction of evidence, many studies have compared broad age bands, national team categories, or non-adjacent performance tiers which provide us with a limited understanding of the capacities that distinguish adjacent competitive categories [9,17,19]. The recent meta-analysis also confirmed heterogeneity in testing methods and performance classification, while a recent systematic scoping review found a lack of research consistently examining multiple age groups besides an evidence-based geographic underrepresentation of South American players [9,20]. Prominent Brazilian studies have focused on national team players, so the extent to which their findings apply to club pathways, where training exposure, selection, and organizational conditions may differ, is underexplored [7,19,20]. A multivariate analysis across consecutive club stages could therefore clarify whether the same physical qualities distinguish U14 from U16, U16 from U18, and U18 from professional players.

The present study therefore compared SJ, CMJ, 20 m sprint, 20 m change-of-direction test (COD-20m), and Yo-Yo IRL1 performance among U14, U16, U18, and professional Brazilian female football players and identified the physical capacities showing the greatest differences between adjacent competitive stages. It was hypothesized that the physical-performance profile would differ across stages, with players at later stages generally demonstrating better physical performance [9,16].

## 2. Materials and Methods

### 2.1. Participants

This analytical cross-sectional study included 119 outfield female football players from the U14 (*n* = 24), U16 (*n* = 19), U18 (*n* = 43), and professional (*n* = 33) competitive stages of two high-level Brazilian clubs. The U14 players were 13.54 ± 0.59 years old, had a body mass of 54.29 ± 7.36 kg, and a stature of 161.17 ± 6.02 cm. The corresponding values were 15.16 ± 0.76 years, 59.81 ± 6.34 kg, and 167.05 ± 4.14 cm for U16 players; 17.31 ± 0.72 years, 60.51 ± 7.18 kg, and 165.56 ± 6.45 cm for U18 players; and 25.48 ± 5.33 years, 60.90 ± 7.51 kg, and 165.21 ± 6.78 cm for professional players. The sample comprised forwards (*n* = 30), midfielders (*n* = 47), center-backs (*n* = 23), and full-backs (*n* = 19). The U14 and U16 groups were composed of players from one club. The U18 group included 22 players from one club and 21 from the other, whereas the professional group included 16 and 17 players from the respective clubs. Players were recruited through convenience sampling according to their participation in the clubs’ pre-season physical-performance assessments. To be included, players were required to belong to the main squad of their respective competitive stage, participate in state or national competitions, and have complete data for the five physical tests analyzed. For the purposes of this study, the professional group comprised players belonging to the main professional squads of their respective clubs and competing in the A1 Division of the Brazilian Women’s Football Championship, the top national division of Brazilian women’s football. Goalkeepers were excluded because of the specific physical and technical demands of their playing position. All 119 eligible outfield players were included in the final analysis, with no additional exclusions and no missing data for the physical-performance tests. The study was conducted in accordance with the principles of the Declaration of Helsinki. Participants were informed about the procedures, potential risks, and benefits before data collection. Written informed consent was obtained from adult participants and from the legal guardians of underage participants, and written assent was obtained from the underage participants. The study was approved by the Research Ethics Committee of the Federal University of Rio Grande do Sul (protocol code 6.894.870; date of approval: 18 June 2024).

### 2.2. Design and Setting

Data were collected during the pre-season period in the regular training environments of the participating clubs. All competitive stages were assessed during the same general phase of pre-season, approximately two weeks after the beginning of the teams’ training programs and following approximately 15 training sessions. Players had approximately 20 h of recovery between their last team training session and the physical assessments. Testing was conducted at 9:00 a.m. and was incorporated into the clubs’ regular physical-performance monitoring routines to minimize interference with training activities. Each competitive category was assessed in a separate morning testing session during its respective pre-season period, with each player completing the full testing battery within a single session. The 20 m sprint, COD-20m, and Yo-Yo IRL1 were performed on natural-grass pitches, with players wearing football boots. Players were instructed to maintain their habitual dietary routines before testing. The same general testing sequence, equipment, verbal instructions, recovery periods, and criteria for selecting the final performance outcome were adopted across the competitive stages.

### 2.3. Procedures

Before testing, the objectives and general procedures were explained to the players. Body mass and stature were assessed before the physical-performance tests. Players subsequently completed a standardized warm-up consisting of 5 min of dynamic stretching followed by 5 min of movements resembling the actions required during the testing battery. The assessments were performed in the following order: anthropometric measurements, squat jump (SJ), countermovement jump (CMJ), 20 m linear sprint, 20 m change-of-direction test (COD-20m), and Yo-Yo Intermittent Recovery Test Level 1 (Yo-Yo IRL1). SJ and CMJ were conducted within the same vertical-jump testing block. A minimum 5 min passive recovery interval was provided between the main physical-testing blocks, during which players rested without performing structured exercise. Recovery intervals between repeated attempts and between the two jump tests are described in the corresponding subsections. For SJ, CMJ, 20 m sprint, and COD-20m, the best result recorded across three attempts was retained for analysis. For the Yo-Yo IRL1, which was performed once, the total distance completed was used as the performance outcome. Sprint and COD performance were analyzed as time in seconds; therefore, lower values represented better performance.

#### 2.3.1. Anthropometric Assessment

Body mass and stature were the anthropometric variables assessed. Body mass was obtained with players barefoot using a digital scale with 0.1 kg resolution (G-TECH, Accumed Produtos Médico Hospitalares Ltd.a., Duque de Caxias, Brazil). Stature was determined while players remained barefoot and stood upright with their backs against a flat wall, using a non-extensible measuring tape fixed vertically to the wall with a resolution of 1 mm. The resulting measurements were expressed in kilograms for body mass and centimeters for stature.

#### 2.3.2. Vertical Jump Test

Lower-limb vertical-jump performance was evaluated with the squat jump (SJ) and countermovement jump (CMJ). Jump height was recorded using a contact mat (Jump System, Cefise, Nova Odessa, Brazil) linked to a portable computer. Throughout both tests, players kept their hands on their hips to restrict the contribution of the upper limbs. For the SJ, each trial began from a stationary semi-squat position with the knees flexed to approximately 90°. Following an auditory command, the player performed a maximal vertical jump without any preliminary countermovement. For the CMJ, the player started from an upright stance and, after the auditory command, rapidly descended to approximately 90° of knee flexion before immediately extending the lower limbs and jumping maximally [21,22]. Three trials were completed for each jump condition, with 1 min of passive recovery separating successive attempts. Once the three SJ trials had been completed, a 3 min recovery period preceded the first CMJ trial. Jump height was derived from flight time according to the equation h = g × t^2^/8, where h denotes jump height, g gravitational acceleration, and t flight time. For each test, the greatest height achieved across the three trials was selected for analysis and reported in centimeters.

#### 2.3.3. Linear Sprint Tests

Linear acceleration was evaluated over 20 m using a maximal sprint protocol. Running time was measured with an electronic timing system (CEFISE, São Paulo, Brazil) equipped with photocells at the start and finish, providing a temporal resolution of 1 ms. Both photocells were positioned 100 cm above the ground. Before each trial, players adopted a standardized split stance with the front foot located 0.30 m behind the initial timing gate. The sprint began after an auditory signal, and players were instructed to accelerate maximally and continue running through the full 20 m distance. To discourage premature deceleration, a cone was placed 5 m beyond the finish line [23,24]. Each player completed three trials, separated by 5 min of passive recovery. The shortest recorded time was selected as the performance outcome and expressed in seconds.

#### 2.3.4. Change-of-Direction Test

Change-of-direction ability was evaluated using the 20 m COD test (COD-20m). The course comprised four consecutive 5 m sections arranged in a zig-zag pattern, with cones defining directional changes of approximately 100°. Players were required to pass around the outside of each cone and complete the entire course in the shortest possible time. Performance was recorded with an electronic timing system (CEFISE, São Paulo, Brazil), using photocells positioned at the start and finish lines 100 cm above the ground. Before each trial, the front foot was placed 0.30 m behind the initial photocell. An additional cone located 5 m beyond the finish line was used to discourage early deceleration before crossing the final timing gate [23,24]. Three trials were completed, with 5 min of passive recovery between attempts. The shortest recorded time was selected for analysis and reported in seconds.

#### 2.3.5. Yo-Yo Intermittent Recovery Test Level 1

Players completed the Yo-Yo Intermittent Recovery Test Level 1 (Yo-Yo IRL1) to evaluate intermittent running performance. The protocol required repeated 20 m out-and-back shuttle runs (2 × 20 m) at running speeds that increased progressively according to audio signals. Following each running bout, players completed 10 s of active recovery within a 2 × 5 m area. Cones delineated both the 20 m shuttle course and the recovery zone. Players were required to maintain the externally prescribed pace for as long as possible. Testing ended when a player voluntarily stopped because of exhaustion or when she failed on two consecutive occasions to reach the required line in time with the audio signal [12]. The Yo-Yo IRL1 was administered once to each player, and the total distance completed was used as the performance outcome and reported in meters.

### 2.4. Statistical Analysis

Descriptive statistics are presented as mean ± standard deviation. Data distribution was examined using the Shapiro–Wilk test, homogeneity of variances using Levene’s test, and homogeneity of covariance matrices using Box’s M test. Given the unequal group sizes and departures from normality identified for some variables, global comparisons of SJ, CMJ, 20 m sprint, 20 m COD, and Yo-Yo IRL1 across competitive stages were performed using Welch’s one-way analysis of variance. When a significant global effect was identified, Games–Howell post hoc tests were used for all pairwise comparisons between competitive stages. Differences in the combined physical-performance profile were examined using multivariate analysis of variance, with competitive stage as the fixed factor and the five physical tests as dependent variables. Pillai’s trace was used as the multivariate statistic. In addition to the overall comparison, three prespecified pairwise multivariate comparisons were performed between adjacent competitive stages: U14 versus U16, U16 versus U18, and U18 versus professional. The resulting three *p*-values were adjusted using the Holm procedure. Potential redundancy among the dependent variables was examined using Pearson’s correlation coefficient. Given the high correlation between SJ and CMJ, the multivariate analysis was repeated using a reduced battery comprising CMJ, 20 m sprint, 20 m COD, and Yo-Yo IRL1. To identify the specific physical capacities that differed between adjacent stages, Welch’s independent-samples *t*-tests were performed separately for U14 versus U16, U16 versus U18, and U18 versus professional players. Mean differences were calculated as the later competitive stage minus the earlier stage and are presented with 95% confidence intervals. Within each adjacent-stage comparison, the five *p*-values were adjusted using the Holm procedure. Hedges’ g, corrected for small-sample bias, was calculated with approximate 95% confidence intervals. Confidence intervals were not adjusted for multiple comparisons and were interpreted alongside the Holm-adjusted *p*-values. Sensitivity analyses were conducted using linear models adjusted for playing position, with heteroscedasticity-consistent HC3 standard errors. The three adjacent-stage comparisons were estimated for each physical test, and the Holm correction was applied to the five outcomes within each comparison. Because the U18 and professional groups included players from both participating teams, an additional comparison between these stages was adjusted simultaneously for playing position and team. Team was not included in analyses involving all competitive stages because the U14 and U16 groups included players from only one team. All tests were two-sided, with statistical significance set at α < 0.05. All analyses were performed using Jamovi software, version 2.6.26.

## 3. Results

Table 1 presents performance in the five physical tests and the global comparisons across competitive stages. Welch’s ANOVA indicated a significant overall effect of competitive stage for all five variables (all *p* < 0.001), and Games–Howell post hoc comparisons were therefore used to identify the specific between-stage differences. Games–Howell post hoc comparisons indicated that, for SJ and CMJ, U14 and U16 players did not differ from each other, whereas U18 players performed better than both younger groups but worse than professional players. In the 20 m sprint, professional players recorded lower times than U14 and U16 players, while the U18 group did not differ significantly from any of the other stages. In the 20 m COD test, professional players recorded lower times than all three youth groups, which did not differ from one another. In the Yo-Yo IRL1, U14 players covered a shorter distance than U16, U18, and professional players, with no differences among the latter three groups.

When the five physical tests were considered simultaneously, a significant multivariate effect of competitive stage was observed. Pairwise multivariate comparisons between adjacent stages also indicated significant differences between U14 and U16, U16 and U18, and U18 and professional players after Holm adjustment. SJ and CMJ were strongly correlated (r = 0.925, *p* < 0.001); therefore, the multivariate analyses were repeated using a reduced battery that retained only CMJ among the two jump tests. In the reduced battery, the overall multivariate effect and all three adjacent-stage comparisons remained significant, as shown in Table 2.

The planned comparisons between adjacent competitive stages are presented in Table 3. Between U14 and U16, only the Yo-Yo IRL1 remained significant after Holm adjustment, with U16 players covering, on average, 491.82 m more than U14 players. Between U16 and U18, SJ and CMJ remained significant after Holm adjustment, with higher values in the U18 group. The 20 m COD test differed before adjustment for multiple comparisons but did not remain significant after Holm correction. Between U18 and professional players, SJ, CMJ, and 20 m COD remained significant after Holm adjustment. Within this comparison, the largest effect was observed for the 20 m COD test, with professional players completing the test, on average, 0.24 s faster. The 20 m sprint did not remain significant after Holm adjustment (*p* = 0.053), and the Yo-Yo IRL1 did not differ significantly between the two stages.

**Figure 1:**
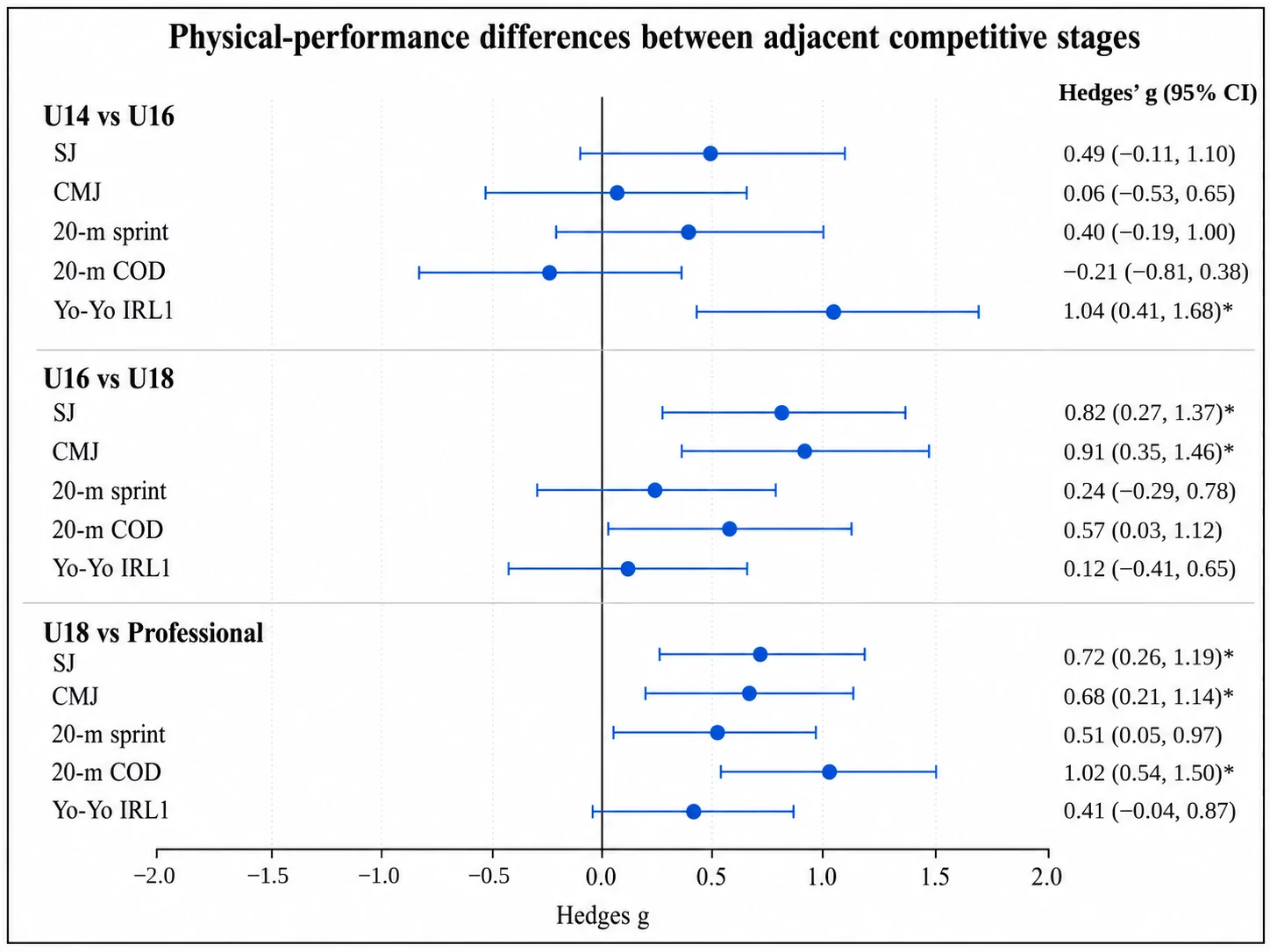
Hedges’ g effect sizes for physical-performance differences between adjacent competitive stages. Points represent Hedges’ g, and horizontal lines represent the corresponding unadjusted 95% confidence intervals. Effects were oriented so that positive values indicate better performance at the later competitive stage. The direction of the 20 m sprint and 20 m COD effects was reversed because lower times represent better performance. The vertical line at zero indicates no standardized difference between stages. The horizontal axis was fixed from −2 to +2. * indicate comparisons with Holm-adjusted *p* < 0.05. CI = confidence interval; U14 = under-14; U16 = under-16; U18 = under-18; SJ = squat jump; CMJ = countermovement jump; COD = change of direction; Yo-Yo IRL1 = Yo-Yo Intermittent Recovery Test Level 1.

Figure 1 presents the effect sizes and their corresponding 95% confidence intervals for the comparisons between adjacent competitive stages. The horizontal axis was fixed from −2 to +2 to allow direct comparison of the magnitude and precision of the effects. Asterisks identify comparisons that remained significant after Holm adjustment.

In the sensitivity analyses using linear models adjusted for playing position, the overall pattern was largely unchanged. Between U14 and U16 players, only the Yo-Yo IRL1 was significant (Holm-adjusted *p* = 0.005). Between U16 and U18 players, SJ and CMJ were significant (Holm-adjusted *p* = 0.004 and 0.001, respectively). Between U18 and professional players, SJ, CMJ, 20 m sprint, and 20 m COD were significant (Holm-adjusted *p* = 0.010, 0.012, 0.043, and <0.001, respectively), whereas the Yo-Yo IRL1 was not significant (Holm-adjusted *p* = 0.074). After additional adjustment for playing position and team in the U18–professional comparison, SJ, CMJ, and 20 m COD remained significant (Holm-adjusted *p* = 0.020, 0.021, and <0.001, respectively), whereas the 20 m sprint was no longer significant (Holm-adjusted *p* = 0.056) and the Yo-Yo IRL1 remained non-significant (Holm-adjusted *p* = 0.094). Overall, the sensitivity analyses supported differences in the Yo-Yo IRL1 between U14 and U16 players, in SJ and CMJ between U16 and U18 players, and in SJ, CMJ, and 20 m COD between U18 and professional players. The 20 m sprint result in the U18–professional comparison was sensitive to adjustment for team. Within this comparison, the largest unadjusted standardized effect was observed for 20 m COD (Hedges’ g = 1.019).

## 4. Discussion

This study aimed to compare physical performance among U14, U16, U18, and professional Brazilian female football players and to identify the physical capacities showing the greatest differences between adjacent competitive stages. The combined physical-performance profile differed across all stages, although the capacities distinguishing each adjacent-stage comparison were not the same. Between U14 and U16, the main difference was observed in intermittent running performance. Between U16 and U18, the clearest differences occurred in vertical-jump performance. Between U18 and professional players, differences were identified in vertical-jump and change-of-direction performance, with the latter showing the greatest magnitude. Linear sprint performance did not remain different after adjustment for multiple comparisons, and intermittent running performance did not distinguish U18 from professional players. These findings demonstrate that physical-performance differences across competitive categories are category- and capacity-specific. Therefore, the hypothesis was supported: the physical-performance profile differed across stages and later-stage players generally presented better performance, although the specific capacities contributing to these differences varied according to the adjacent-stage comparison examined.

Our findings agree with the broader evidence that higher performance standards in women’s football are generally accompanied by better intermittent fitness, sprint performance, and lower-limb power, although the magnitude of the differences varies substantially according to the test protocol and the way competitive level is classified [9,25]. Results from a meta-analysis reported moderate-to-large between-tier differences in Yo-Yo IRL1 distance, 20 and 30 m sprint time, and SJ and CMJ height, while also identifying considerable heterogeneity across studies and competitive classifications [9]. A cross-sectional study including 414 high-level female players aged 12–21 years old similarly showed age-related improvements in sprint, jump, and agility performance, but suggested that better results became smaller after approximately 16 years of age [17]. A longitudinal study of a female national team program further showed that maximal speed, CMJ, broad-jump performance, and intermittent fitness improved through the youth years, whereas trajectories were not identical across capacities and changed around the transition into the early twenties [16]. Our results extend this evidence by examining adjacent-stage contrasts within a common testing framework. Our results are interesting since consecutive stages could be differentiated by the combined profile even when only one or two individual tests survived multiplicity adjustment, indicating that no single physical capacity adequately represents the entire competitive pathway.

The comparison between U14 and U16 was mainly characterized by a large difference in Yo-Yo IRL1 performance, whereas the jump, sprint, and COD measures did not provide statistically significant discrimination. This finding aligns with the results from a meta-analysis which showed that intermittent field tests show some of the largest differences across the women’s football performance scale [9]. The Yo-Yo IR tests assess the capacity to repeatedly perform intense shuttle running with brief recovery periods and impose substantial aerobic loading together with a meaningful anaerobic contribution [12,26]. In elite female football, performance in a related Yo-Yo intermittent test has also been shown to vary by competitive standard and to improve following concentrated training, supporting its sensitivity to both player level and training status [27]. The approximately 492 m difference observed here should not be interpreted as evidence of age-related or developmental change and may therefore reflect a combination of maturation, accumulated football exposure, conditioning history, category-specific training, and selection within the sampled club. In elite youth female players, biological maturity has been associated with differences in aerobic capacity, speed, COD, and power-related characteristics, although the pattern is not uniform across adjacent maturity groups [13]. Likewise, a large cross-sectional study of adolescent female players found that age effects on several field-based fitness measures were limited or dependent on whether maturation was controlled [28]. Because maturation status and training history were not measured in the present study, their contribution to both the observed Yo-Yo IRL1 difference and the absence of significant differences in the other physical tests cannot be separated from the effect of competitive category. In the present sample, SJ, CMJ, 20 m sprint, and COD-20m did not differ significantly between U14 and U16 players after adjustment for multiple comparisons.

Between U16 and U18, the clearest differences occurred in SJ and CMJ, with moderate-to-large standardized effects and mean advantages of approximately 3–4 cm in the older group. This tendency follows previous evidence showing progressive improvements in vertical-jump performance across adolescence and into early adulthood in high-level female football [16,17]. Age-related analyses of elite youth female players have attributed better jump performance in older groups mostly to greater power production during standing jumps [29], while a longitudinal study has shown continuing CMJ improvement during the youth phase [16]. The parallel differences in SJ and CMJ, together with their strong correlation in the present sample, can be aligned with a difference in lower-limb vertical force-production capacity rather than an isolated advantage specific to the countermovement. However, jump height derived from flight time does not identify the kinetic or neuromuscular source of the difference. The COD-20m comparison requires careful interpretation. Its effect estimate was moderate, but the result did not survive Holm adjustment, so the data suggest a possible U16-to-U18 difference without providing sufficiently robust evidence to declare one. The lack of an adjusted difference in the 20 m sprint also diverges from a study of U14, U16, and U18 female players in which the older categories were faster across sprint splits [14]. Differences in maturity distribution, training background, category composition, sprint protocol, sample size, and the present use of multiplicity control may justify the differences. Thus, the U16-to-U18 findings indicate that vertical-jump performance was the most clearly differentiated domain, whereas the estimates for sprint and COD were less consistent and should not be interpreted as proof of no between-category difference.

The comparison between U18 and professional players was distinguished by higher SJ and CMJ performance and, most prominently, faster COD-20m performance. The large COD effect is consistent with evidence that multidirectional speed is an important component of the physical profile in high-level female football and is related to, but not interchangeable with, linear speed and lower-body power [25,30,31]. Studies in elite and collegiate female players have shown that change-of-direction performance shares variance with sprint and jump qualities while retaining task-specific demands associated with braking, body reorientation, and reacceleration [30,31]. The observed between-category difference may therefore reflect cumulative exposure to higher-intensity training and match demands, greater neuromuscular development, and selection for players who can repeatedly decelerate and redirect efficiently, although these are just possibilities. Because the present comparison was cross-sectional, these explanations remain hypotheses and require further research. The 20 m sprint did not differ significantly between U18 and professional players after Holm adjustment. The literature generally shows faster sprint performance at higher standards and in older high-level female players [9,17]; however, the present comparison did not meet the predefined statistical significance threshold after adjustment for multiple comparisons. The Yo-Yo IRL1 performance also did not differ significantly between U18 and professional players. This differs from research using the Yo-Yo intermittent endurance level 2 test, in which elite senior women are better than elite youth players, but the test variant, group definitions, and competitive contexts were not directly comparable [27]. Accordingly, test selection should be considered when interpreting this finding, as the use of Yo-Yo IRL1 may have limited the discriminatory sensitivity between U18 and professional players; however, a ceiling effect was not directly assessed in the present study. A study in national-level players indicates that intermittent fitness can continue to improve during the youth phase before its age trajectory changes in early adulthood, which may partly explain why U18 players in a high-level pathway can approach senior values on a field test [16]. Moreover, Yo-Yo IRL1 distance should be interpreted as performance in a standardized intermittent running task rather than as a direct estimate of maximal oxygen uptake or a complete representation of match-running capacity [6,32]. Thus, the non-significant U18-to-professional Yo-Yo result indicates that these groups were not statistically differentiated by this specific test in the present sample, but it does not establish equivalent aerobic physiology or equivalent readiness for professional match demands.

This study has limitations that should be considered when interpreting the findings. Its cross-sectional design does not allow the observed differences to be interpreted as individual changes caused by progression through the competitive stages. The sample was obtained by convenience from two high-level Brazilian clubs, and club representation was not balanced across categories because U14 and U16 players came from one club, whereas U18 and professional players came from both. Although sensitivity analyses adjusted for playing position and, when possible, team, residual effects related to club-specific training, selection, and competitive contexts cannot be excluded, and the U14–U16 comparison should therefore be interpreted within the specific context of the sampled club. Biological maturation, training history, and recent internal or external training loads were not assessed, which may be particularly relevant when comparing youth categories, especially the U14 and U16 groups. The unequal group sizes, particularly the smaller U16 group, may also have reduced the precision of estimates for smaller between-category differences. Although standardized recovery intervals were provided between testing blocks, some residual fatigue before the final Yo-Yo IRL1 cannot be completely excluded. Ambient temperature and relative humidity were not systematically recorded across testing sessions and therefore represent an additional limitation when interpreting the findings. The study also included only outfield players and a limited set of physical tests. Therefore, the findings should not be generalized to goalkeepers or interpreted as representing technical, tactical, psychological, or overall football performance.

Despite these limitations, the study provides a detailed comparison of four consecutive competitive stages using the same field-based testing battery within an applied pre-season context. The combination of global, multivariate, and prespecified adjacent-stage analyses, together with effect sizes and sensitivity models, allowed the differences to be examined beyond isolated group means. In practice, the findings can help coaches and performance staff interpret physical assessments according to the adjacent-stage comparison being considered: intermittent running performance showed the clearest difference between U14 and U16, vertical-jump performance between U16 and U18, and vertical-jump and change-of-direction performance between U18 and professional players. These results should be used as complementary reference points rather than isolated selection criteria because competitive advancement in football remains multifactorial. Future longitudinal and multicenter studies should track players across categories, incorporate biological maturation and training exposure, and combine physical measures with technical, tactical, psychological, and match-performance indicators.

## 5. Conclusions

Physical performance differed among U14, U16, U18, and professional Brazilian female football players, but the capacities distinguishing adjacent competitive stages were not uniform. Intermittent running performance primarily differentiated U14 from U16 players within the sampled club, vertical-jump performance differentiated U16 from U18 players, and vertical-jump and change-of-direction performance differentiated U18 from professional players. Change-of-direction performance showed the greatest difference between U18 and professional players, whereas linear sprint and intermittent running performance did not independently distinguish these groups after adjustment for multiple comparisons. These findings indicate that the physical characteristics distinguishing competitive categories in women’s football vary according to the adjacent-stage comparison examined. Therefore, we suggest that assessments be interpreted within the specific competitive context of each category. In practice, coaches and performance staff may use these category-specific findings as complementary reference points to guide physical monitoring and training decisions across competitive stages in women’s football.

## Figures and Tables

**Table 1:** Physical performance across competitive stages, Welch’s ANOVA, and Games–Howell post hoc comparisons.

Variable	U14 (*n* = 24)	U16 (*n* = 19)	U18 (*n* = 43)	Professional (*n* = 33)	Welch’s F (df_1,_ df_2_)	*p*
SJ (cm)	23.38 ± 3.71 ^a^	25.05 ± 2.71 ^a^	28.15 ± 4.09 ^b^	31.05 ± 3.78 ^c^	24.044 (3, 57.31)	<0.001
CMJ (cm)	24.50 ± 3.21 ^a^	24.69 ± 3.27 ^a^	28.56 ± 4.57 ^b^	31.50 ± 3.91 ^c^	23.286 (3, 57.10)	<0.001
20 m sprint (s)	3.44 ± 0.14 ^a^	3.39 ± 0.11 ^a^	3.35 ± 0.14 ^ab^	3.29 ± 0.12 ^b^	6.722 (3, 55.08)	<0.001
20 m COD (s)	5.58 ± 0.26 ^a^	5.63 ± 0.22 ^a^	5.49 ± 0.26 ^a^	5.25 ± 0.19 ^b^	18.257 (3, 53.93)	<0.001
Yo-Yo IRL1 (m)	962.92 ± 378.20 ^a^	1454.74 ± 550.83 ^b^	1512.09 ± 430.53 ^b^	1695.76 ± 448.96 ^b^	16.075 (3, 52.76)	<0.001

Note: Values are presented as mean ± standard deviation. Within each row, means with different superscript letters differ significantly according to the Games–Howell post hoc test (*p* < 0.05). Superscript letters a, b, and c denote statistically homogeneous groups: means sharing at least one letter do not differ significantly, whereas means with no letter in common differ significantly. Welch’s F is the test statistic from Welch’s one-way analysis of variance; df1 and df2 are the numerator and Welch–Satterthwaite denominator degrees of freedom, respectively. Lower values indicate better performance in the 20 m sprint and 20 m COD tests. *n* = number of players; U14 = under-14; U16 = under-16; U18 = under-18; SJ = squat jump; CMJ = countermovement jump; COD = change of direction; Yo-Yo IRL1 = Yo-Yo Intermittent Recovery Test Level 1.

**Table 2:** Multivariate comparisons of physical performance across competitive stages.

Test battery	Comparison	Pillai’s Trace	F	df_1_	df_2_	*p*	Holm-Adjusted *p*
Five-test battery	Overall competitive-stage effect	0.665	6.431	15	339	<0.001	—
Five-test battery	U14 vs. U16	0.420	5.348	5	37	<0.001	0.003
Five-test battery	U16 vs. U18	0.181	2.472	5	56	0.043	0.043
Five-test battery	U18 vs. Professional	0.231	4.195	5	70	0.002	0.004
Reduced battery	Overall competitive-stage effect	0.624	7.486	12	342	<0.001	—
Reduced battery	U14 vs. U16	0.392	6.129	4	38	<0.001	0.002
Reduced battery	U16 vs. U18	0.181	3.145	4	57	0.021	0.021
Reduced battery	U18 vs. Professional	0.230	5.314	4	71	<0.001	0.002

Note: The five-test battery included SJ, CMJ, 20 m sprint, 20 m COD, and Yo-Yo IRL1. The reduced battery included CMJ, 20 m sprint, 20 m COD, and Yo-Yo IRL1. Pillai’s trace is the multivariate test statistic, and F is the corresponding approximate F statistic; df_1_ and df_2_ are the numerator and denominator degrees of freedom, respectively. The Holm adjustment was applied to the three adjacent-stage comparisons within each test battery and was not applicable to the overall omnibus effect, represented by an em dash. U14 = under-14; U16 = under-16; U18 = under-18; SJ = squat jump; CMJ = countermovement jump; COD = change of direction; Yo-Yo IRL1 = Yo-Yo Intermittent Recovery Test Level 1.

**Table 3:** Planned comparisons between adjacent competitive stages.

Comparison	Variable	Mean Difference (95% CI)	Welch’s t (df)	*p*	Holm-Adjusted *p*	Hedges’ g (95% CI)
U14 vs. U16	SJ (cm)	1.67 (−0.31, 3.65)	1.70 (40.78)	0.096	0.386	0.49 (−0.11, 1.10)
U14 vs. U16	CMJ (cm)	0.19 (−1.83, 2.20)	0.19 (38.43)	0.854	0.945	0.06 (−0.53, 0.65)
U14 vs. U16	20 m sprint (s)	−0.05 (−0.13, 0.03)	−1.37 (40.89)	0.177	0.532	0.40 (−0.19, 1.00)
U14 vs. U16	20 m COD (s)	0.05 (−0.09, 0.20)	0.73 (40.76)	0.473	0.945	−0.21 (−0.81, 0.38)
U14 vs. U16	Yo-Yo IRL1 (m)	491.82 (189.64, 794.00)	3.32 (30.61)	0.002	0.012	1.04 (0.41, 1.68)
U16 vs. U18	SJ (cm)	3.10 (1.33, 4.87)	3.52 (50.50)	<0.001	0.004	0.82 (0.27, 1.37)
U16 vs. U18	CMJ (cm)	3.87 (1.81, 5.93)	3.78 (47.31)	<0.001	0.002	0.91 (0.35, 1.46)
U16 vs. U18	20 m sprint (s)	−0.03 (−0.10, 0.04)	−0.96 (41.67)	0.343	0.686	0.24 (−0.29, 0.78)
U16 vs. U18	20 m COD (s)	−0.14 (−0.27, −0.02)	−2.26 (40.83)	0.030	0.089	0.57 (0.03, 1.12)
U16 vs. U18	Yo-Yo IRL1 (m)	57.36 (−234.28, 348.99)	0.40 (28.15)	0.690	0.690	0.12 (−0.41, 0.65)
U18 vs. Professional	SJ (cm)	2.89 (1.09, 4.70)	3.20 (71.46)	0.002	0.008	0.72 (0.26, 1.19)
U18 vs. Professional	CMJ (cm)	2.93 (0.99, 4.88)	3.01 (73.08)	0.004	0.011	0.68 (0.21, 1.14)
U18 vs. Professional	20 m sprint (s)	−0.07 (−0.13, −0.01)	−2.26 (72.11)	0.027	0.053	0.51 (0.05, 0.97)
U18 vs. Professional	20 m COD (s)	−0.24 (−0.34, −0.14)	−4.63 (73.92)	<0.001	<0.001	1.02 (0.54, 1.50)
U18 vs. Professional	Yo-Yo IRL1 (m)	183.66 (−20.05, 387.37)	1.80 (67.49)	0.076	0.076	0.41 (−0.04, 0.87)

Note: Mean differences were calculated as the later competitive stage minus the earlier stage. Negative mean differences in the 20 m sprint and 20 m COD indicate lower times and, therefore, better performance at the later stage. Welch’s t is the test statistic from Welch’s independent-samples *t*-test, and df represents the corresponding Welch–Satterthwaite degrees of freedom. The Holm adjustment was applied separately to the five physical tests within each adjacent-stage comparison. Hedges’ g is the standardized mean difference corrected for small-sample bias and was oriented so that positive values consistently indicate better performance at the later stage; therefore, its direction was reversed for the 20 m sprint and 20 m COD. The 95% confidence intervals were not adjusted for multiple comparisons and should be interpreted alongside the Holm-adjusted *p*-values. Bold values indicate comparisons with Holm-adjusted *p* < 0.05. CI = confidence interval; U14 = under-14; U16 = under-16; U18 = under-18; SJ = squat jump; CMJ = countermovement jump; COD = change of direction; Yo-Yo IRL1 = Yo-Yo Intermittent Recovery Test Level 1.

## Data Availability

The data presented in this study are available from the corresponding author upon reasonable request. The data are not publicly available because of privacy and ethical restrictions related to the potential identification of the participating female football players.
